# Effect of Ultrasound Pretreatment on the Moisture Migration and Quality of *Cantharellus cibarius* Following Hot Air Drying

**DOI:** 10.3390/foods12142705

**Published:** 2023-07-14

**Authors:** Mianli Sun, Yuan Xu, Yangyue Ding, Ying Gu, Yongliang Zhuang, Xuejing Fan

**Affiliations:** Faculty of Food Science and Engineering, Kunming University of Science and Technology, No. 727 South Jingming Road, Kunming 650500, China; 20212114045@stu.kust.edu.cn (M.S.); 20212214082@stu.kust.edu.cn (Y.X.); dingyangyue77@163.com (Y.D.); guying@kust.edu.cn (Y.G.); ylzhuang@kmust.edu.cn (Y.Z.)

**Keywords:** ultrasound pretreatment, *Cantharellus cibarius*, hot air drying, moisture migration, antioxidant activity

## Abstract

The effect of different ultrasound pretreatment powers (0–500 W) before hot air drying on the moisture migration and quality of *Cantharellus cibarius* (*C. cibarius*) was investigated in this study. The results showed that the ultrasound pretreatment accelerated the drying rate. When the ultrasound power was 400 W, the drying time of *C. cibarius* was reduced by 18.90% compared with the control group. The low-field nuclear magnetic resonance (LF-NMR) and magnetic resonance imaging (MRI) results showed that the ultrasound pretreatment increased the water mobility in *C. cibarius*. The scanning electron microscopy (SEM) results revealed that the ultrasound pretreatment promoted the expansion of intercellular pores. In addition, the rehydration capacity and quality characteristics of the ultrasound-pretreated dried *C. cibarius* were better than those of the control group. Overall, this study concluded that ultrasound pretreatment is a promising pretreatment method for the hot air drying of *C. cibarius* products to reduce the total drying time significantly and improve the retention rate of the total phenolics and flavonoids of dried *C. cibarius*.

## 1. Introduction

*Cantharellus cibarius (C. cibarius)*, one of the most popular edible chanterelle mushrooms, is a highly appreciated foodstuff due to its special apricot flavor, its richness in nutrients, and its low fat content [1]. In particular, the phenolic substances (such as catechin, pyrogallol, myricetin, phenolic and protocatechuic acids) contained in *C. cibarius* make its total antioxidant content superior to that of any mushroom studied [2]. It can be used fresh, dried, or processed as pickled products. Although fresh *C. cibarius* has a more delicious and unique flavor [1], its shelf life is short and it rots easily due to its high water activity, high water content, and lack of a hard outer layer to protect it from physical and microbial attacks [3]. Moreover, the fresh *C. cibarius* harvesting season is limited and expensive. Therefore, it is necessary to take some measures to extend the shelf life of fresh *C. cibarius* and maintain its nutritional quality as much as possible. Drying is a widely used processing technology in mushroom preservation that prevents spoilage by reducing the moisture content to a level that allows safe storage. Many techniques have been described for mushroom drying, such as hot air drying, microwave drying, freeze drying, and sun drying [4].

Hot air drying technology is used to dry edible mushrooms because of its simple operation and low cost. However, a longer drying time leads to the degradation of heat-sensitive compounds and the loss of characteristic flavor compounds [5]. Moreover, improper temperature control would also cause product shrinkage, reduce rehydration capacity, and destroy nutrients [6]. Nowadays, with the increasing demand for high-quality foods, traditional hot air drying is unable to meet the needs of consumers [7]. To overcome the traditional drying process’s limitations and produce high-quality products, it is necessary to explore an effective pretreatment method.

The pretreatment process before thermal drying can be divided into chemical pretreatment (liquid phase and gas phase) and physical pretreatment (thermal and non-thermal pretreatment) [8]. As a chemical pretreatment, osmotic pretreatment has the advantage of improving the drying process and maintaining product quality [8]. Pei et al. observed that osmotic dehydration accelerates moisture diffusion during drying and shortens the drying time [9]. However, in the case of a highly concentrated osmosis solution, material migration leads to the loss of minerals, vitamins, and pigments. Similarly, although blanching as a physical pretreatment can effectively inactivate various enzymes and destroy micro-organisms, thereby improving quality and promoting the drying speed, it will cause changes in product quality, soluble nutrients, pigments and flavors. Ultrasound, as a non-thermal food processing technique, can improve drying efficiency and inactivate enzymes while avoiding the detrimental effects of heat on the nutritional and sensory properties of foods compared to thermal processing [10,11]. Ultrasonic pretreatment is increasingly being used in the food industry as a non-thermal food processing technique (drying, thawing, extraction) [10,12,13].

In addition, the ultrasonic waves propagate through the material, causing a series of rapid alternating compression expansion and cavitation cycles, resulting in microchannels within the sample. Water molecules can diffuse to the surface through the microscopic channels, increasing the effective water diffusivity [14]. Ultrasound pretreatment before hot air drying has been found to significantly enhance the drying efficiency of mushrooms over untreated samples and to reduce the loss of nutritious substances [15,16]. Furthermore, ultrasonication causes the rehydration capacity of the drying products to increase [17]. Low-field nuclear magnetic resonance (LF-NMR) and magnetic resonance imaging (MRI) have become powerful tools for studying the dynamic behavior of water in food processing due to their high efficiency, low cost, and non-destructive nature. LF-NMR is used to study the mobility and distribution of water, while MRI images are used to observe the internal distribution of water and changes during the drying process [18]. However, there is limited research on the effect of different ultrasonic pretreatment powers on moisture migration and *C. cibarius* quality.

In this study, different ultrasonic powers were applied before hot air drying to evaluate the impact of ultrasound pretreatment on the overall quality of dried *C. cibarius*. Moreover, the moisture migration during drying was analyzed using the LF-NMR and MRI techniques. An effective pretreatment method is presented according to the comprehensive assessment, which is designed to improve the drying characteristics and quality of *C. cibarius*.

## 2. Materials and Methods

### 2.1. Raw Materials

Fresh *C. cibarius* mushrooms were purchased from the local wild mushroom market in Kunming, Yunnan (China), with an initial water content of 91.65 ± 0.01 g/100 g. The raw materials were wrapped in plastic and stored in a refrigerator at 4 °C to prevent water loss and ensure the quality of the materials. Folin-Ciocalteu’s phenol reagent, aluminum nitrate, sodium nitrate, 2, 2-diphenyl-1-picrohydrazyl were purchased from Aladdin Biochemical Technology Co., Ltd. (Shanghai, China). All chemicals used were of analytical grade.

### 2.2. Ultrasound Pretreatment

The *C. cibarius* was pretreated by ultrasound in an Ultrasonic Cell Disruptor (SCIENTZ-IID, Ningbo Xinzhi Biotechnology Co., Ltd., Ningbo, China). *C. cibarius* (40 ± 1 g) and deionized water (240 mL) were placed in a beaker and maintained at 15 ± 1 °C using an ice bath [19]. The ultrasonic pretreatment power was set at 0, 100, 200, 300, 400, and 500 W for 20 min.

### 2.3. Hot Air Drying

The pretreated samples were weighed and dried in a hot air dryer (101 A-1 E, Shanghai Experimental Instrument Factory Co., Ltd., Shanghai, China) at 60 ± 1 °C until reaching a constant weight. To analyze the moisture content of the mushrooms during drying, the samples were weighed every 30 min.

#### 2.3.1. Measurement of Moisture Ratio

The moisture ratio (MR) was calculated according to Equation (1) [20]:

(1) $$\mathrm{MR}=\frac{m_{t}-m_{e}}{m_{0}-m_{e}}$$

where m_t_, m_0_, and m_e_ are the moisture content, initial moisture content, and equilibrium moisture content (g water/g dry matter) at any time, respectively. For longer drying times, the value of m_e_ is small compared to m_t_ or m_0_. Therefore, Equation (1) can be simplified to Equation (2):

(2) $$\mathrm{MR}=\frac{m_{t}}{m_{0}}$$

#### 2.3.2. Measurement of Drying Rate

The drying rate (DR) was calculated using Equation (3):

(3) $$\mathrm{DR}=\frac{m_{t}-m_{t+\Delta t}}{\Delta t}$$

where $m_{t}$ and $m_{t+\Delta t}$ are the moisture content at t and t + Δt (g water/g dry matter), respectively; moreover, t is the time (min) and Δt is the time difference (min) [21].

### 2.4. LF-NMR Measurement

The transverse relaxation times (T_2_) of the samples with different pretreatments were determined using an LF-NMR analyzer (Niumag, Suzhou, Jiangsu, China). The T_2_ of each sample was measured via scanning with a Carr–Purcell–Meiboom–Gill (CPMG) pulse sequence. The 90° and 180° pulse durations were 20.0 μs and 40.0 μs, respectively. The time waiting TW = 3000 ms, the number of echoes NECH was 7000, and the cumulative number Ns was 4 [22].

### 2.5. MRI Measurement

The ultrasound-pretreated *C. cibarius* samples were dried with hot air for 0, 90, and 180 min. The internal moisture distribution of the samples was examined via MRI at the above times. The MRI image analysis parameter settings were: time echo (TE): 20 ms, repetition time: 500 ms, and 256 × 256 matrix. Proton density-weighted imaging was performed using the SE sequences [22].

### 2.6. Scanning Electron Microscopy (SEM)

The microstructure of the pileus and stipe of the samples subjected to different ultrasound power pretreatments was observed via SEM (Tescan Mira4+ Energy Spectrum xplore 30 EDS, TESCAN Trading Co., Ltd., Kohoutovice, Czech Republic) according to Li et al., with small modifications [15]. A thin section was taken from each dried sample, sputter-coated with gold, and examined via SEM. Images of the pileus and stipes were analyzed at 5000× and 500× magnifications, respectively.

### 2.7. Color Determination

The color parameters of the samples were analyzed using a colorimeter [23] (NR110, Shenzhen 3NH Technology Co., Ltd., Shenzhen, China). The total chromatic aberration (ΔE) was calculated by Equation (4):

(4) $$\Delta E=\sqrt{{\left(L^{*}-{L_{0}}^{*}\right)}^{2}+{\left(a^{*}-{a_{0}}^{*}\right)}^{2}+{\left(b^{*}-{b_{0}}^{*}\right)}^{2}}$$

where *L*_0_*, *a*_0_*, and *b*_0_* are the color values of the fresh product and L*, a*, and b* are the color values of the dry product.

### 2.8. Rehydration Ratio

The rehydration ratio of the dried *C. cibarius* was determined according to Zhao et al., with small modifications [24]. A quantity of dried *C. cibarius* pretreated with different ultrasound powers was selected and immersed in distilled water at a temperature maintained at 40 °C and then weighed every one hour until a constant weight was reached. The rehydration rate (RR) was obtained from Equation (5):

(5) $$\mathrm{RR}=\frac{m_{f}}{m_{g}}$$

where $m_{f}$ is the weight of the rehydrated samples and m_g_ is the weight of the initial dried samples (g).

### 2.9. Determination of Bioactive Substances and Antioxidant Activities

The dried *C. cibarius* samples were crushed and passed through a 40-mesh sieve. Then, they were extracted via ethanol-assisted sonication.

#### 2.9.1. Antioxidant Activity (DPPH)

The DPPH free radical-scavenging activity of the sample extracts was determined according to the method of Wang et al., with minor modifications [25]. The DPPH was then calculated using Equation (6):

(6) $$\mathrm{DPPH}\;\mathrm{Radical}\;\mathrm{Scavenging}\;\mathrm{Activity}\;\%=\frac{1-\left(A_{i}-A_{j}\right)}{A_{0}}\times 100$$

where A_i_, A_0_, and A_j_ are the absorbance of the DPPH solution when adding the antioxidant, without the antioxidant and control, respectively.

#### 2.9.2. Total Phenolics Content (TPC)

The phenolic content of the mushroom was estimated using the Folin–Ciocalteau assay described by Bao et al., with minor modifications [26]. The results were expressed as the mg of gallic acid equivalents (GAE) per 100 g of dry weight (DW).

#### 2.9.3. Total Flavonoid Compounds

The total flavonoid content of the mushroom was measured using the aluminum chloride colorimetric assay described by Konieczynski et al., with slight modifications [27]. The results were expressed as the mg of rutin equivalent (RE) per 1 g dry weight (DW).

### 2.10. Statistical Analysis

All the analyses were repeated at least three times, and the data were expressed as the mean ± standard deviation. An analysis of variance (ANOVA, Tukey’s test) was performed to determine whether the data showed significant differences within the same group. Statistic for Windows (Version 8.1, Analytical Software, St Paul, MN, USA) was used for the analysis. A significance level of 0.05 was used.

## 3. Results and Discussion

### 3.1. Drying Characteristics of C. cibarius

**Figure 1:**
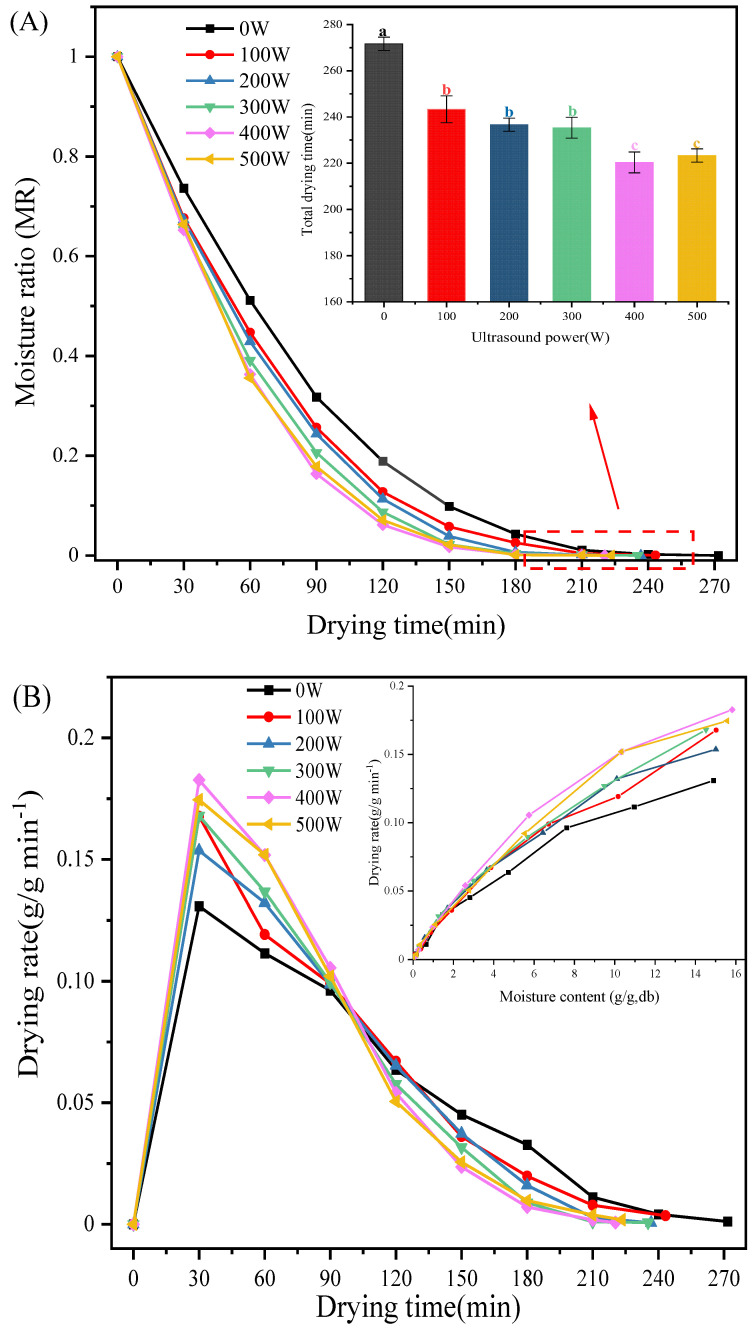
(**A**) Effects of the ultrasound pretreatments on the moisture ratio (MR)—time profiles and final moisture content of *C. cibarius* after hot air drying. (**B**) Drying rate (DR) versus drying time curve for mushrooms with different drying pretreatments under hot air drying (60 °C). Different letters indicate significant difference (*p* < 0.05); Same letter means no significant difference (*p* > 0.05).

The drying rate and moisture ratio of *C. cibarius* pretreated with different ultrasound powers during the hot air drying process are depicted in Figure 1. As shown in Figure 1A, the moisture ratio decreased exponentially with increasing drying time. Compared with the control group, the total drying time decreased significantly with increasing ultrasound power (*p* < 0.05). When the ultrasound power reached 400 W, the moisture ratio decreased the fastest and the drying time dropped significantly from 271.67 min (control group) to only 220.33 min (*p* < 0.05). These results indicated that the ultrasound pretreatment had a positive effect on the drying of *C. cibarius*. A similar decreasing tendency of drying time with the application of ultrasonic pretreatment was reported in the convective drying of apple slices [28], heat pump drying of scallops [11] and pulsed fluidized bed microwave freeze drying of Chinese yam [29]. This could be due to the cavitation effect of the ultrasound treatment and the rapid shrinkage and expansion of the sample, which promote the formation of microchannels within the sample and facilitate the diffusion of water during the drying process [19]. However, there were no significant differences in the total drying time between the 400 W and 500 W ultrasound treatment groups. This could be because the higher ultrasound power further destroyed the internal structure of the *C. cibarius*. Ni et al. also reported that pretreatment with different ultrasound powers resulted in different effects on the drying process of goji berries [30].

As shown in Figure 1B, before drying for 30 min, the drying rate of all the groups showed a sharp upward trend, and then it showed a downward trend with the increase in drying time. The drying rate of *C. cibarius* pretreated with different ultrasound powers was higher than that of the control group in the first 90 min. Furthermore, the highest drying rate was investigated in the 400 W ultrasound pretreatment group. These results indicated that the ultrasound pretreatment resulted in rapid moisture removal in the initial phase of drying and increased the drying rate. Similarly, the acceleration of the drying rate was also due to the fact that the ultrasonic treatment changed the microstructure of the sample and promoted water loss during the drying process, thus increasing the drying rate [19]. After 120 min of drying, the drying rate of the control group was higher than that of the other groups, while the drying rate of the other groups gradually decreased. This is due to the fact that the ultrasonic treatment accelerated the removal of free water during the drying of the samples, while the remaining bound and fixed water was not sensitive to hot air drying. In contrast, the samples in the blank group had free water throughout the hot air drying process, resulting in a higher drying rate at a later stage than in the pretreatment group [31].

### 3.2. LF-NMR Analysis of C. cibarius

**Figure 2:**
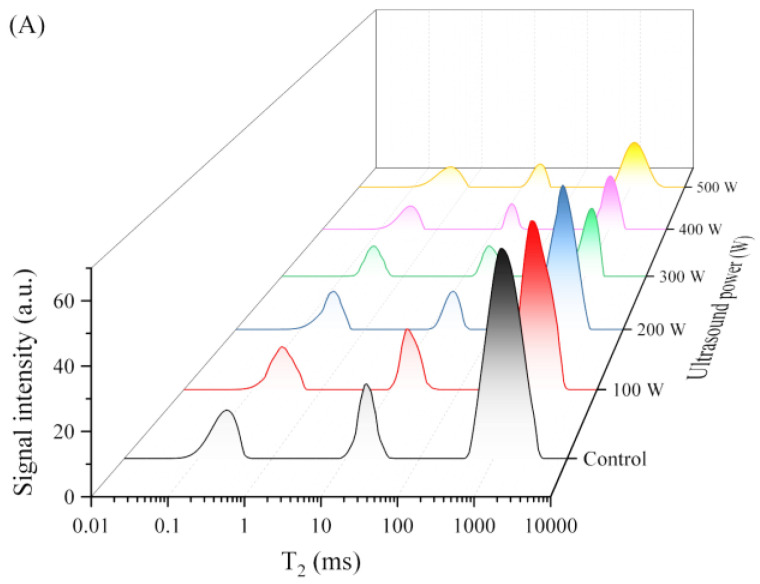

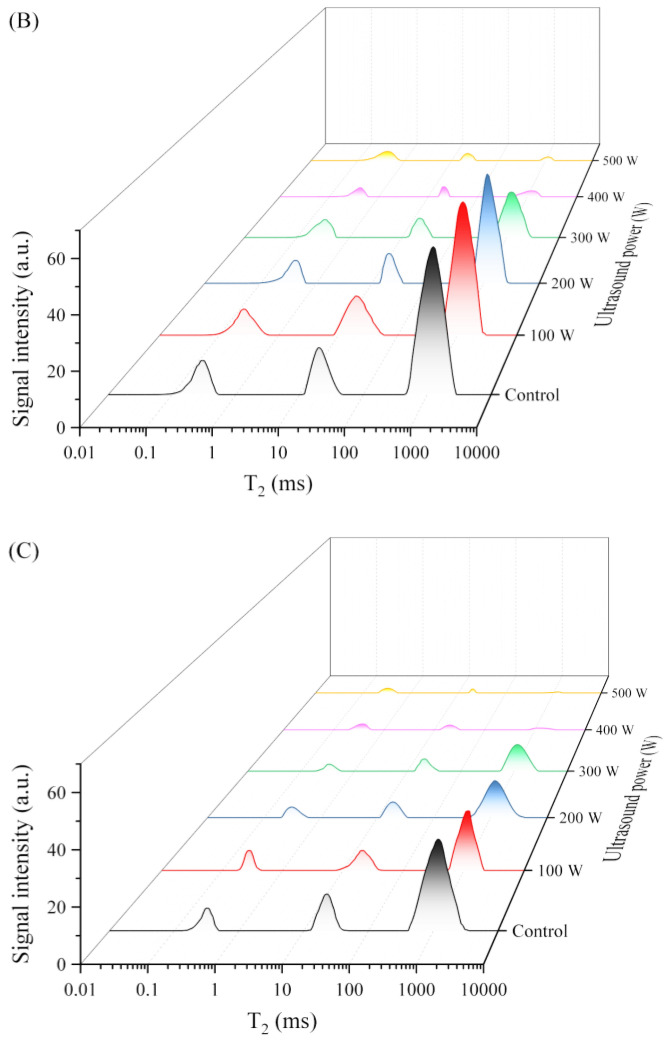
T_2_ relaxation spectra of *C. cibarius* during drying with different ultrasonic pretreatments: (**A**) 0, (**B**) 90, and (**C**) 180 min.

To investigate the migration rate and distribution of water molecules, and to understand the effect of ultrasound pretreatment on the water status of *C. cibarius* tissue during the drying process, the transverse relaxation time (T_2_) of the samples dried for 0, 90, and 180 min was measured using LF-NMR (Figure 2). A total of three peaks were revealed in the T_2_ curves of the *C. cibarius* samples: T_21_ (0.01–1 ms), T_22_ (1–100 ms), and T_23_ (100–10,000 ms). The water molecules of T_21_, T_22_, and T_23_ are defined as bound water, immobilized water, and free water, respectively [32]. The length of the relaxation time is closely related to the degree of freedom of water. The longer the relaxation time, the higher the degree of freedom of water and the easier its removal. Hence, the order of these three forms of moisture removed from the samples during the drying process was as follows: T_21_ < T_22_ < T_23_ [33]. Compared to the untreated group, the water distribution in the ultrasonically treated samples changed. It is possible that ultrasound alters the internal structure of the chanterelles, leading to a redistribution of water within the cells. A similar situation was found in a study of water changes during the microwave vacuum drying of Lotus (*Nelumbo nucifera Gaertn.*) [34] and infrared drying of potato chips [35] after ultrasonic pretreatment.

As shown in Figure 2, with increasing drying time, the T_2_ values of all the samples decreased significantly, which indicates that water was gradually lost. This was consistent with the moisture ratio trend of *C. cibarius* in the drying process. Compared with the control group, the ultrasound pretreatment significantly affected the water distribution in the samples. As the ultrasonic power increased, the signal strength of the sample T_2_ relaxation curve decreased continuously, especially the signal strength of T_23_. This suggests that the degree of freedom and the amount of free water decrease during drying, while higher drying temperatures also accelerate changes in water mobility and distribution [22]. These results show that the microchannels formed by the ultrasonic pretreatment reduce the resistance to water diffusion during drying, promote water flow, and accelerate water loss [36]. When the drying time reached 90 min, the signal intensity of T_23_ in the samples pretreated by ultrasound at 400 and 500 W decreased significantly. Moreover, the signal intensities of T_21_ and T_22_ were very weak. When the drying time reached 180 min, there was little signal intensity of T_23_ in the samples subjected to ultrasound pretreatment at 400 and 500 W. Only a few signal intensities of T_21_ and T_22_ were present. These results were consistent with the change in the drying rate, suggesting that the samples had dried almost to a constant weight and that there was a little water, mainly in the form of bound water [37]. Moreover, the smaller the proportion of free water in the entire drying process and in the final dry product, the better the drying effect. Zhang et al. have also found that the water loss was mainly from free water and immobile water during the drying process [18].

### 3.3. MRI Analysis of Drying of C. cibarius

**Figure 3:**
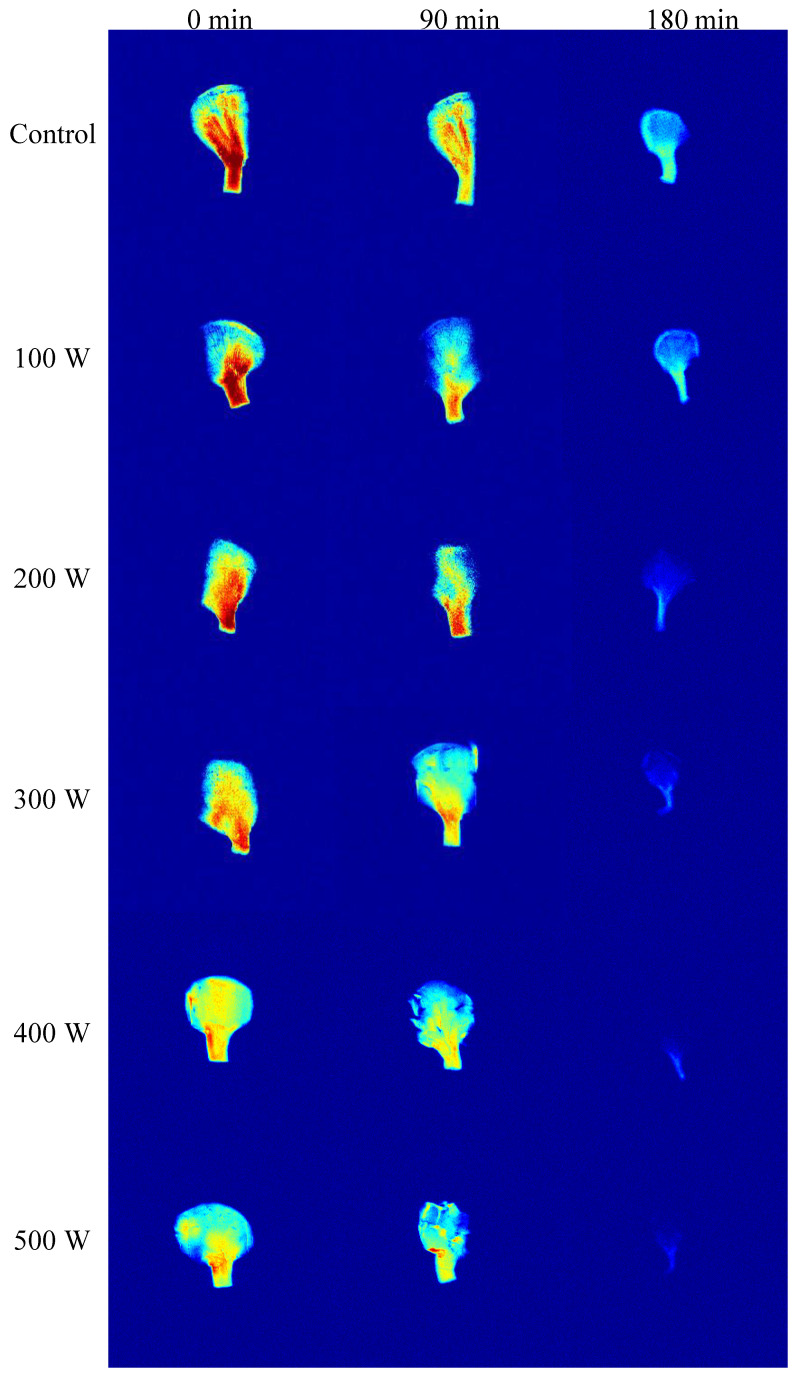
MRI of *C. cibarius* during drying under different ultrasonic pretreatments.

**Figure 4:**
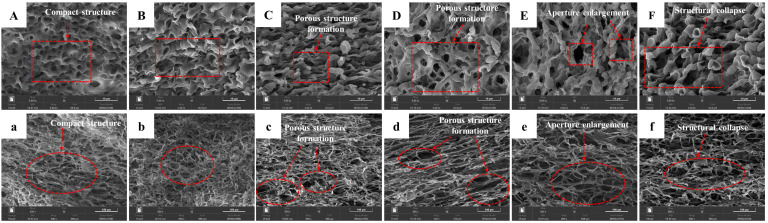
Microstructure of dried *C. cibarius* pileus under different ultrasound power pretreatment conditions: (**A**) control, (**B**) 100 W, (**C**) 200 W, (**D**) 300 W, (**E**) 400 W, and (**F**) 500 W. Microstructure of the longitudinal section of dried *C. cibarius* stipe under different ultrasonic power pretreatment conditions: (**a**) control, (**b**) 100 W, (**c**) 200 W, (**d**) 300 W, (**e**) 400 W, and (**f**) 500 W.

Figure 3 shows MRI images of *C. cibarius* pretreated by ultrasound with different powers during the drying process. In the MRI images, the number of mobile hydrogen atoms is proportional to the color. The red color indicates high water content and high signal intensity, and the blue color indicates the opposite [38]. As shown in Figure 3, the water distribution in the sample was affected by the ultrasonic pretreatment. The moisture in the fresh samples before drying was unevenly distributed, and the water content in the stipe was higher than that in the pileus. With the prolongation of the drying time, the water content decreased from the central part toward the epidermis, which was mainly due to the dehydration of the surface of the samples and drying shrinkage [22]. Compared with the control group, the water content in the ultrasound-pretreated samples decreased significantly in the drying process. After drying for 90 min, it can be seen that the imaging signal inside the sample was weakened, which proves that the moisture content was reduced. Especially when the ultrasonic power reached 400 and 500 W, the obtained imaging signal strength was poor. When the drying time was 180 min, the proton signal of the control group was still high. However, there was almost no proton signal in the images of the samples pretreated with ultrasound (400 and 500 W). This phenomenon was caused by the fact that the ultrasound pretreatment increased the convective mass transfer and promoted the evaporation of water in the hot air drying process [39]. At the same time, in the process of hot air drying, the external water of the chanterelles was removed by the influence of hot air, while the internal water continued to migrate to the outside [32]. These results were similar to the T_2_ relaxation curve of the samples during the drying process. Additionally, the decrease in the water content in the sample also led to a decrease in the drying rate in the later drying period (Section 3.1). The scanning electron microscopy results also confirmed that the microstructure of the control group hindered water diffusion (Figure 4).

### 3.4. Effect of Different Ultrasonic Pretreatment Powers on Sample Microstructure

The effect of the different ultrasound pretreatment powers on the microstructure of hot air-dried *C. cibarius* is shown in Figure 4. Although a honeycomb ultra-structure and pores were observed in all the samples, significant differences in the microstructure were also observed between the untreated and treated samples. As seen in Figure 4A,a, the microstructure of the pileus and stipe of the control group was compact. After the ultrasound pretreatment with different powers, the intercellular space and pores expanded, and the intercellular space of the samples was more evident with the increase in ultrasound power until the ultrasound power reached 400 W. This was due to the formation of microchannels and the larger honeycomb structure caused by acoustic cavitation during the ultrasound pretreatment process [35]. Similarly, the formation of microchannels in Pleurotus eryngii was promoted by the high-frequency vibration and cavitation of ultrasonic waves [19]. In a study involving the ultrasonic treatment of potato chips, it was found that with the increase in the ultrasonic pretreatment power, more porous cell structures were formed inside the dry potato chips [35]. When the ultrasound power was 500 W, the internal pore structures of the pileus showed partial collapse of the cellular tissue. This might be because the frequent ultrasonic vibration with high power caused contractions and expansions, which caused the sample to repeatedly squeeze and release [24]. The study by Zhang et al. also found that high-power ultrasound destroyed the microstructure of the sample, which adversely affected the finished product [40]. At the same time, applying ultrasonic waves before drying may increase the structural damage caused by temperature rises during drying [41]. Therefore, the structure of the untreated samples could hinder the outward diffusion of moisture in the drying process. The large pore size and intercellular space in the ultrasound-pretreated samples increased the water fluidity in the tissue and the drying rate, decreasing the drying time. Similarly, Zhao et al. found that ultrasound application changed the microstructure of shiitake mushrooms and increased the water loss rate during the drying process [24]. Moreover, ultrasound pretreatment also reduced the drying time of kiwifruit, sweet potatoes, celery, and strawberries by increasing the porous structure and promoting the diffusion of internal water [42].

### 3.5. Effect of Different Ultrasonic Pretreatment Powers on the Color of Samples 

Color is one of the critical parameters of dried products, which determines market value and consumer acceptance [28]. The L*, a*, b*, and ΔE values of *C. cibarius*, including the pileus and stipe, are listed in Table 1. Compared to fresh samples, the L* values of all the dried samples decreased significantly. The decreased L* values of the dried samples could be attributed to the long Maillard reaction time during the drying process [43]. In the dried samples, the L* values of the control group were lower than those of the ultrasound-pretreated group. This could be because the ultrasound pretreatment reduced the drying time, which avoided adverse effects on product quality due to prolonged drying. In a study on the color of potato chips after ultrasonic pretreatment, Wu et al. also found that the brightness of the potato chips after ultrasonic pretreatment increased and was higher than that of the fresh samples [35]. With increasing ultrasound power, the L* value increased and then decreased. When the ultrasound power was 400 W, the L* value was closest to that of the fresh samples. This indicates that although the hot air drying process caused enzymatic browning of the product, the 400 W treatment significantly reduced the drying time of the mushrooms and did not cause severe enzymatic browning [32]. However, the L* value of the dried samples pretreated by ultrasound at 500 W decreased significantly. This may be because the 500 W ultrasonic treatment disrupted the cells, causing the release of the enzymes responsible for browning on the surface and making the samples look dark [44].

Compared to the fresh samples, the a* and b* values increased, indicating that the redness and yellowness increased during the drying process due to sample pigmentation. After the ultrasound pretreatment, the a* and b* values were lower than those of the control group. This phenomenon could be attributed to the enzymatic browning of the samples with a long drying process. The a* and b* values decreased and then increased with increasing ultrasound power. When the ultrasound power was 400 W, the a* and b* values decreased to the lowest level. Moreover, there was no significant difference between the fresh pileus and ultrasound-pretreated pileus at 400 W. These results indicate that the ultrasound pretreatment at 400 W had the least influence on the color of the dried *C. cibarius*. Similarly, the study by Shi et al. found that the brightness of dried shiitake mushroom slices was increased by ultrasonic pretreatment, which maintained the brightness of the dried mushroom slices [45]. Çakmak et al. made a similar discovery in relation to the drying of mushrooms [17].

### 3.6. Effect of Different Ultrasonic Pretreatment Powers on the Rehydration Ratio of the Samples

In this study, the effect of ultrasound pretreatment with different powers on the rehydration ratio (RR) of dried *C. cibarius* was investigated (Table 2). The results showed that the RR of the ultrasound-pretreated group was significantly higher than that of the control group (5.73) (*p* < 0.05). Similarly, Zhu et al. also found that the rehydration rate of dried scallops pretreated with ultrasound was higher than that of dried scallops without pretreatment [11]. The RR first increased significantly and then decreased slightly with increasing ultrasound power. When the ultrasound power was 400 W, the RR reached a maximum (6.11) and the sample showed excellent water absorption ability. The poor rehydration ability of the dried samples in the control group could be due to the decrease in the size of the available pores in the tissue during the long drying process and the irreversible shrinkage of the tissue structure caused by drying [46]. The ultrasound pretreatment not only reduced the drying time (Section 3.1) and shrinkage but also promoted the formation of a porous microstructure that increased the moisture absorption capacity during the rehydration process [24]. In addition, the RR slightly decreased when the ultrasound power was 500 W. This could be because the high power pretreatment caused the mushroom to form a porous structure, and at the same time, part of the inner wall collapsed, reducing the rehydration capacity [44]. Jarahizadeh and Dinani used different ultrasonic powers to treat potato chips prior to convection drying and found that increasing the ultrasonic power reduced the rehydration capacity of potato chip dry products [47].

### 3.7. Effect of Different Ultrasonic Pretreatment Powers on Nutritional Quality

**Figure 5:**
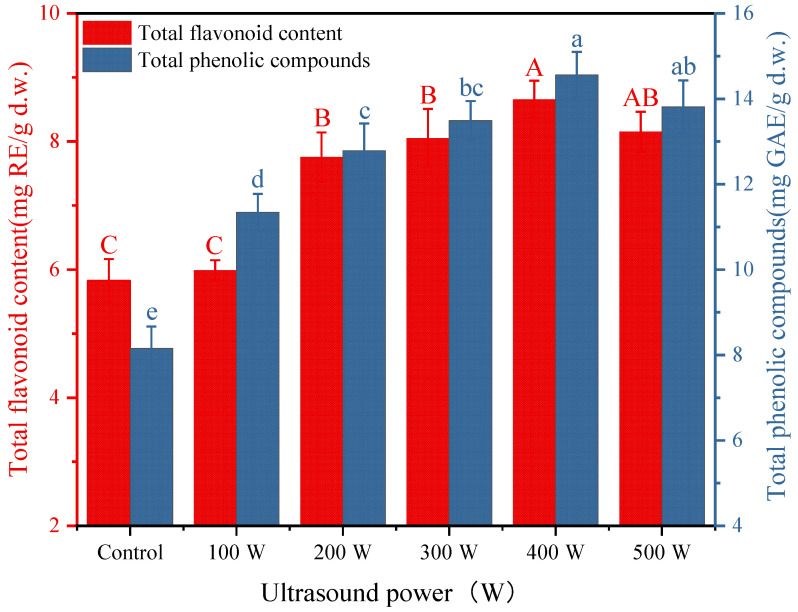
Total phenolics and flavonoids contents of dried *C. cibarius* pretreated with different ultrasound powers. Different letters indicate significant difference (*p* < 0.05). Capital letters indicate significance analysis for total flavonoid content and small letters indicate significance analysis for total phenolic compounds.

The contents of total phenolics and flavonoids in dried *C. cibarius* showed a similar trend. As shown in Figure 5, the total phenolics and flavonoids contents of the samples pretreated with different ultrasound powers were higher than those of the control group. With increasing ultrasound power, the contents of phenol and flavonoids increased significantly (*p* < 0.05) and then decreased slightly. These results indicate that the ultrasound pretreatment effectively retained the total phenolics and flavonoids contents of the dried *C. cibarius*. The retention of the total phenolics content could be attributed to the inactivation of enzymes by the ultrasound pretreatment and the inhibition of the polyphenol oxidase-induced oxidation of phenolic compounds [45]. In addition, the long drying time in the control group caused prolonged exposure of the *C. cibarius* to high temperature and oxygen, resulting in strong oxidation and the loss of total phenolic substances [48]. Li et al. also reported that ultrasound pretreatment reduced the loss of phenolic compounds in dried mushroom slices [15]. However, the total phenolics and flavonoids contents of the dried samples decreased slightly under the 500 W ultrasound pretreatment. This might be because the high-power ultrasound pretreatment destroyed the sample cells, while the intracellular compounds diffused into the surrounding medium, resulting in the decreased extraction of phenolic and flavonoid compounds [35].

A similar trend in the antioxidant activity of dried *C. cibarius* pretreated by ultrasound with different powers was also observed (Table 2). Compared to the control group, the antioxidant activity of the ultrasound-pretreated samples increased significantly (*p* < 0.05). With increasing ultrasound power, the antioxidant activity increased at first and then decreased significantly (*p* < 0.05). When the ultrasound power was 400 W, the antioxidant activity reached a maximum (81.61). This could be because the ultrasound pretreatment could have shortened the drying time of the samples, protecting *C. cibarius* from elevated temperature and oxygen damage. In addition, the ultrasound pretreatment could have promoted the release of the non-phenolic antioxidant ergothioneine from the protein structure, resulting in an improvement in the antioxidant properties [49]. Similar results were also found for ultrasonically pretreated dried mushroom slices [21].

## 4. Conclusions

The effects of different ultrasonic pretreatment powers on the water migration of *C. cibarius* and its quality were evaluated. The experimental results of this study showed that ultrasonic pretreatment before hot air drying significantly decreased the total drying time of *C. cibarius* compared to the untreated samples. When the ultrasonic pretreatment power was 400 W, the total drying time was reduced by 18.90%. The ultrasound pretreatment played a positive role in the drying of *C. cibarius*. In particular, the pretreatment at 400 W provided a good condition for improving the retention rate of the DPPH, total phenols and total flavonoids of the dried samples and accelerating the drying rate. The ultrasonic pretreatment formed a porous structure in the mushroom tissue, which accelerated the drying rate, and the dried product had good color and rehydration ability. This study revealed that 400 W ultrasonic pretreatment could be recommended as a drying condition to obtain high-quality dried *C. cibarius*. However, improper temperature control in the ultrasonic pretreatment process will have adverse effects, so it is necessary to further optimize the pretreatment temperature control program to obtain better pretreatment products.

## Figures and Tables

**Table 1:** Color measurement of fresh and dried *C. cibarius* pileus and stipe.

Ultrasound Power	Pileus				Stipe			
	L*	a*	b*	ΔE	L*	a*	b*	ΔE
Fresh	77.62 ± 0.76 ^a^	6.23 ± 0.43 ^e^	73.61 ± 0.42 ^a b^	——	86.58 ± 0.40 ^a^	0.13 ± 0.03 ^b^	10.51 ± 0.72 ^d^	——
Control	66.83 ± 1.18 ^d^	17.64 ± 0.91 ^a^	66.18 ± 3.34 ^d^	17.55 ± 2.03 ^a^	71.40 ± 0.43 ^d^	6.64 ± 0.55 ^a^	30.75 ± 0.51 ^c^	26.14 ± 0.31 ^c,d^
100 W	71.38 ± 0.35 ^c^	7.99 ± 0.64d ^e^	71.84 ± 0.65 ^a,b,c^	6.82 ± 0.59 ^c^	71.44 ± 0.49 ^d^	6.80 ± 0.11 ^a^	31.85 ± 0.99 ^c^	27.08 ± 0.61 ^c^
200 W	71.47 ± 0.35 ^c^	8.49 ± 0.93 ^c^	72.15 ± 3.62 ^a,b,c^	8.59 ± 0.51 ^c^	72.65 ± 0.45 ^c,d^	6.92 ± 1.74 ^a^	38.93 ± 3.42 ^a^	32.43 ± 2.30 ^a^
300 W	71.82 ± 0.38 ^c^	9.52 ± 0.90 ^c,d^	68.83 ± 0.18 ^b,c,d^	8.23 ± 1.12 ^c^	73.53 ± 0.52 ^c^	7.55 ± 1.15 ^a^	35.19 ± 1.08 ^a,b,c^	28.51 ± 1.72 ^b,c^
400 W	75.48 ± 0.10 ^b^	6.99 ± 0.58 ^d,e^	74.89 ± 0.87 ^a^	2.65 ± 1.01 ^d^	83.36 ± 0.54 ^b^	1.91 ± 0.29 ^b^	33.06 ± 2.09 ^b,c^	22.87 ± 1.77 ^d^
500 W	68.16 ± 0.53 ^d^	14.61 ± 1.53 ^b^	67.98 ± 0.66 ^c,d^	13.95 ± 0.60 ^b^	71.34 ± 1.00 ^d^	7.95 ± 1.92 ^a^	37.58 ± 1.57 ^a,b^	32.09 ± 0.62 ^a b^

Different letters in the same column indicated significant difference (*p* < 0.05); The same letter indicates no significant difference (*p* > 0.05).

**Table 2:** Effect of different ultrasound power pretreatments on the moisture diffusion coefficient, rehydration ratio (g/g) and antioxidant activity (%) of *C. cibarius*.

Ultrasound Power	Rehydration Ratio (g/g)	Antioxidant Activity (%)
Control	5.73 ± 0.14 ^c^	77.76 ± 0.53 ^c^
100 W	5.87 ± 0.13 ^b,c^	78.61 ± 0.36 ^c^
200 W	5.99 ± 0.13 ^a,b^	80.11 ± 0.24 ^b^
300 W	6.00 ± 0.08 ^a,b^	80.97 ± 0.96 ^a,b^
400 W	6.11 ± 0.16 ^a^	81.61 ± 0.86 ^a^
500 W	5.99 ± 0.07 ^a,b^	80.90 ± 0.46 ^a,b^

Different letters in the same column indicated significant difference (*p* < 0.05); The same letter indicates no significant difference (*p* > 0.05).

## Data Availability

The data used to support the findings of this study can be made available by the corresponding author upon request.
